# An RNAi-Based Control of *Fusarium graminearum* Infections Through Spraying of Long dsRNAs Involves a Plant Passage and Is Controlled by the Fungal Silencing Machinery

**DOI:** 10.1371/journal.ppat.1005901

**Published:** 2016-10-13

**Authors:** Aline Koch, Dagmar Biedenkopf, Alexandra Furch, Lennart Weber, Oliver Rossbach, Eltayb Abdellatef, Lukas Linicus, Jan Johannsmeier, Lukas Jelonek, Alexander Goesmann, Vinitha Cardoza, John McMillan, Tobias Mentzel, Karl-Heinz Kogel

**Affiliations:** 1 Institute for Phytopathology, Centre for BioSystems, Land Use and Nutrition, Justus Liebig University, Giessen, Germany; 2 Institute of General Botany and Plant Physiology, Friedrich-Schiller-University, Jena, Germany; 3 Institute for Microbiology and Molecular Biology, Centre for BioSystems, Land Use and Nutrition, Justus Liebig University, Giessen, Germany; 4 Institute of Biochemistry, Centre for BioSystems, Land Use and Nutrition, Justus Liebig University, Giessen, Germany; 5 Institute for Bioinformatics and Systems Biology, Centre for BioSystems, Land Use and Nutrition, Justus Liebig University, Giessen, Germany; 6 BASF Plant Science LP, Research Triangle Park, Durham, North Carolina, United States of America; 7 BASF SE, Limburgerhof, Germany; University of California, Davis Genome Center, UNITED STATES

## Abstract

Meeting the increasing food and energy demands of a growing population will require the development of ground-breaking strategies that promote sustainable plant production. Host-induced gene silencing has shown great potential for controlling pest and diseases in crop plants. However, while delivery of inhibitory noncoding double-stranded (ds)RNA by transgenic expression is a promising concept, it requires the generation of transgenic crop plants which may cause substantial delay for application strategies depending on the transformability and genetic stability of the crop plant species. Using the agronomically important barley—*Fusarium graminearum* pathosystem, we alternatively demonstrate that a spray application of a long noncoding dsRNA (791 nt *CYP3*-dsRNA), which targets the three fungal cytochrome P450 *lanosterol C-14α-demethylases*, required for biosynthesis of fungal ergosterol, inhibits fungal growth in the directly sprayed (local) as well as the non-sprayed (distal) parts of detached leaves. Unexpectedly, efficient spray-induced control of fungal infections in the distal tissue involved passage of *CYP3*-dsRNA via the plant vascular system and processing into small interfering (si)RNAs by fungal DICER-LIKE 1 (*FgDCL-1*) after uptake by the pathogen. We discuss important consequences of this new finding on future RNA-based disease control strategies. Given the ease of design, high specificity, and applicability to diverse pathogens, the use of target-specific dsRNA as an anti-fungal agent offers unprecedented potential as a new plant protection strategy.

## Author Summary

RNA interference has emerged as a powerful genetic tool for scientific research. The demonstration that agricultural pests, such as insects and nematodes, are killed by exogenously supplied RNA targeting their essential genes has raised the possibility that plant predation can be controlled by lethal RNA signals. We show that spraying barley with a 791 nt long dsRNA (*CYP3*-dsRNA) targeting the three fungal ergosterol biosynthesis genes (*CYP51A*, *CYP51B*, *CYP51C*), whose respective proteins also are known as azole fungicide targets, efficiently inhibited the necrotrophic fungus *Fusarium graminearum* in directly sprayed and systemic leaf tissue. Strong inhibition of fungal growth required an operational fungal RNA interference mechanism as demonstrated by the fact that a Fusarium DICER-LIKE-1 mutant was insensitive to *CYP3*-dsRNA in systemic, non-sprayed leaf areas. Our findings will help in the efficient design of RNAi-based plant disease control. We provide essential information on a fundamentally new plant protection strategy, thereby opening novel avenues for improving crop yields in an environmentally friendly and sustainable manner.

## Introduction

According to the FAO [1], more than half of the world’s harvested area is allotted to cereals such as rice, maize and wheat (ca. 2.3 billion tons in 2010). Diseases of cereal crops such as Fusarium head blight (FHB) and Fusarium seedling blight (FSB), caused by necrotrophic fungi of the genus Fusarium, exert a particularly great economic and agronomic impact on global grain production and the grain industry [2,3]. Food safety can be compromised by contamination of agricultural products with mycotoxins, which are produced during FHB and FSB development [4] and represent a serious threat to human and animal health. Currently, the major strategies to control Fusarium diseases include resistance breeding, crop rotation, and biological control along with the application of DMI (demethylation inhibitors) fungicides [5]. DMI fungicides, such as tebuconazole, triadimefon, and prochloraz inhibit ergosterol biosynthesis by binding to cytochrome P450 lanosterol C-14 α-demethylase (CYP51), thereby disrupting fungal membrane integrity [6]. However, heavy reliance on DMI fungicides since their discovery in the mid-1970s holds a risk of the emergence of DMI-tolerant strains of plant pathogens. Conventional plant breeding strategies have been only partly successful, as the quantitative nature of FHB and FSB resistance does not allow straightforward breeding programs.

Since the discovery in 1998 that exogenous double-stranded (ds)RNA triggers suppression of gene activity in a homology-dependent manner [7], along with the identification of small RNAs (sRNAs) as a new class of regulatory molecules [8] that functions via RNA interference (RNAi), our understanding of the essential cellular function of gene silencing has increased considerably [9–10]. Mobile RNA silencing signals are capable of translocating from the host to its interacting organism, and vice versa [11–14]. Recent evidence supports the significant contribution of sRNAs and RNAi to the communication between plant hosts and a pathogenic fungus [15]. Exploiting the RNAi mechanism in plants also has a strong potential for agriculture. Indeed, expression of inhibitory dsRNAs in the corresponding host plant conferred protection from predation or infection by targeted gene silencing [16–18], a phenomenon that has been termed host-induced gene silencing (HIGS).

Recently, we demonstrated that in Arabidopsis (*Arabidopsis thaliana*) and barley (*Hordeum vulgare*), transgenic expression of *CYP3*-dsRNA, a 791 nt long dsRNA targeting the three fungal *CYP51* genes involved in ergosterol biosynthesis, confers resistance to infection with *Fusarium graminearum* [19]. While these results provided proof-of-concept that RNAi-based plant protection is an effective strategy for controlling diseases caused by devastating necrotrophic pathogens, the broad applicability of this transgenic method remains questionable due to the persisting weak acceptance of GMO strategies for food and feed production in many countries. More important, a broad application of this transgenic approach is hampered by the lack of transformability of various crop plants and the missing genetic stability of the silencing trait. Here we investigate the potential and the mechanism of an RNAi-based crop protection strategy using direct spray applications of *CYP3*-dsRNA to target *F*. *graminearum*. We show that the 791 nt long dsRNA is taken up by the plant and transferred in an unmodified form via the vascular system to fungal infection sites where it is processed by the fungal RNAi machinery as a prerequisite for its antifungal activity. We show a strong correlation between accumulation of *CYP3-*dsRNA at infection sites, silencing of *CYP51* expression, and fungal inhibition.

## Results

### Spray-induced gene silencing (SIGS) of Fusarium genes

**Fig 1:**
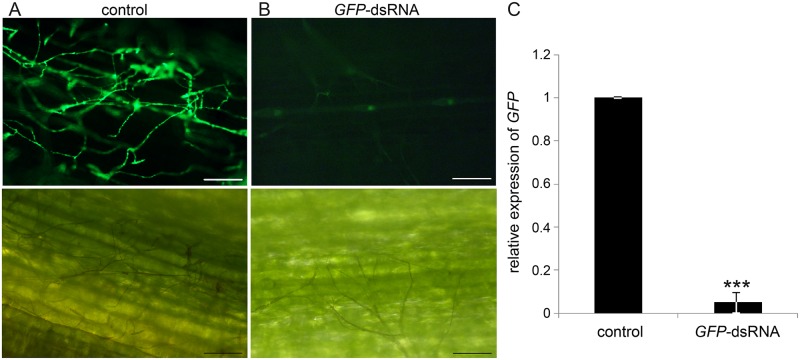
(A-C) Spray-induced gene silencing (SIGS) of *GFP* expression in *Fusarium graminearum* strain Fg-IFA65_GFP_. Detached second leaves of three-week-old barley plants were locally sprayed with Tris-EDTA (TE, **A**, control) or *GFP*-dsRNA **(B)**. Forty-eight hours after spraying, distal, non-sprayed leaf segments were drop-inoculated with Fg-IFA65_GFP_ (20 μL of a solution containing 2 x 10^4^ conidia mL^-1^). *GFP* silencing efficiency was visualized 6 dpi using confocal microscopy. **(C)** *GFP* transcripts were quantified by qPCR at 6 dpi. The reduction in fungal *GFP* expression on leaves sprayed with *GFP*-dsRNA and infected with Fg-IFA65_GFP_ compared with TE-sprayed controls was statistically significant (***P < 0.001; Student´s *t* test). Bars represent mean values ± SDs of three independent experiments. Scale bars represent 100 μm.

To provide a proof of concept, we conducted an experiment targeting the expression of the jellyfish *green fluorescent protein* (*GFP*) in the *GFP*-expressing *F*. *graminearum* strain Fg-IFA65_GFP_ [20] by using a *GFP*-specific 720 nt long dsRNA (*GFP*-dsRNA, S1 Fig). Detached barley leaves were locally sprayed with 20 ng μL^-1^ *GFP*-dsRNA or Tris-EDTA buffer (TE, control) and drop-inoculated 48 h later with Fg-IFA65_GFP_ in the distal (non-sprayed) leaf segment. Confocal microcopy showed strong GFP fluorescence associated with fungal mycelia on TE-treated control leaves at six days post inoculation (dpi) (Fig 1A). In contrast, fluorescence (Fig 1B) and *GFP* transcripts (Fig 1C) were largely absent in mycelia grown on leaves that were locally sprayed with *GFP*-dsRNA, although mycelial growth was unrestricted as evidenced by light microscopy. This observation clearly demonstrates the possibility of targeting a gene of an attacking microbe via SIGS.

**Fig 2:**
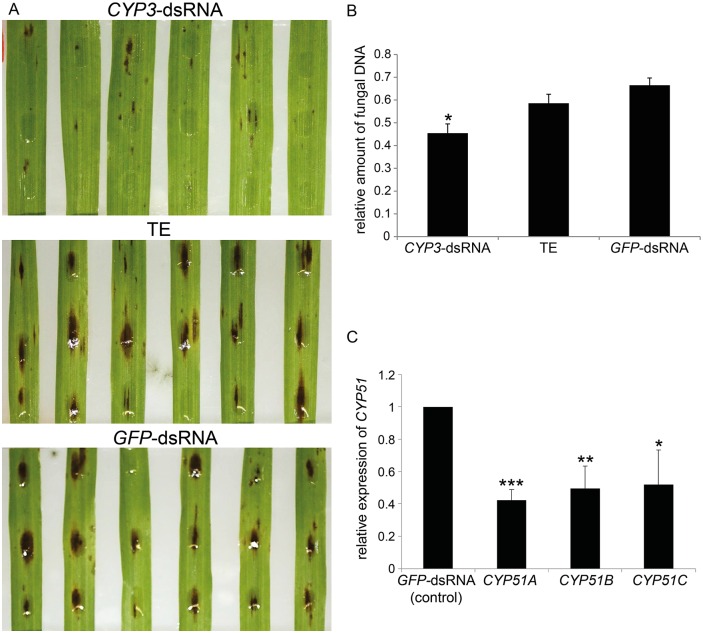
(A-C) SIGS-mediated control of *F*. *graminearum* on leaves sprayed with *CYP3*-dsRNA. **(A)** Detached second leaves of three-week-old barley were sprayed evenly with *CYP3*-dsRNA, TE (mock control), and *GFP*-dsRNA (negative control), respectively. After 48 hours, leaves were drop-inoculated with 2 × 10^4^ conidia mL^−1^ of Fg-IFA65 onto the sprayed area and evaluated for necrotic lesions at 6 dpi. **(B)** The relative amount of fungal DNA at 6 dpi as measured by qPCR was reduced in *CYP3*-dsRNA-treated leaves compared to control leaves. Bars represent mean values ± SDs of three independent experiments. The reduction of fungal growth on *CYP3*-dsRNA vs. TE- or *GFP*-dsRNA-sprayed leaves was statistically significant (*P < 0.05; Student´s t test). **(C)** Gene-specific qPCR analysis of fungal *CYP51A*, *CYP51B*, and *CYP51C* transcripts at 6 dpi (corresponding to 8 d after spraying). The reduction in fungal *CYP51* gene expression on *CYP3*-dsRNA-sprayed leaves as compared with *GFP*-dsRNA-sprayed controls was statistically significant (*P < 0.05, **P < 0.01, ***P < 0.001; Student´s t test).

To further explore the potential of SIGS, we assessed the silencing efficiency of *CYP3*-dsRNA, which targets the three Fusarium genes *CYP51A*, *CYP51B*, and *CYP51C*. The 791 nt long *CYP3*-dsRNA contains complementary fragments of these genes starting with N-terminal *CYP51B*, followed by *CYP51A* and *CYP51*C [19]. Leaves were sprayed with *CYP3*-dsRNA and 48 h later drop-inoculated directly onto the sprayed area with Fg-IFA65. At six dpi, *CYP3*-dsRNA-treated leaves developed brownish lesions that were substantially smaller than those on TE- or *GFP*-dsRNA-sprayed leaves that served as control in this experiment (Fig 2A). Quantitative real-time PCR (qPCR) analysis of fungal DNA levels, based on the ratio between fungal tubulin and plant ubiquitin, confirmed reduced fungal growth on *CYP3*-dsRNA-treated leaves (Fig 2B). To confirm that inhibition of Fusarium growth by *CYP3*-dsRNA was provoked by sequence-specific gene silencing, expression of all the three fungal *CYP51* genes was assessed. At six dpi, total RNA was isolated from infected leaves and the levels of *CYP51A*, *CYP51B* and *CYP51C* transcripts were measured by qPCR and normalized to the expression of the fungal *ß-tubulin* gene. Consistent with the concept of spray-induced gene silencing, we found that the relative amounts of *CYP51* transcripts were reduced on average by 58% (*CYP51A*), 50% (*CYP51B*), and 48% (*CYP51C*) in leaves sprayed with *CYP3*-dsRNA vs. the *GFP*-dsRNA control (Fig 2C).

### SIGS confers strong resistance against Fusarium in distal leaf parts

**Fig 3:**
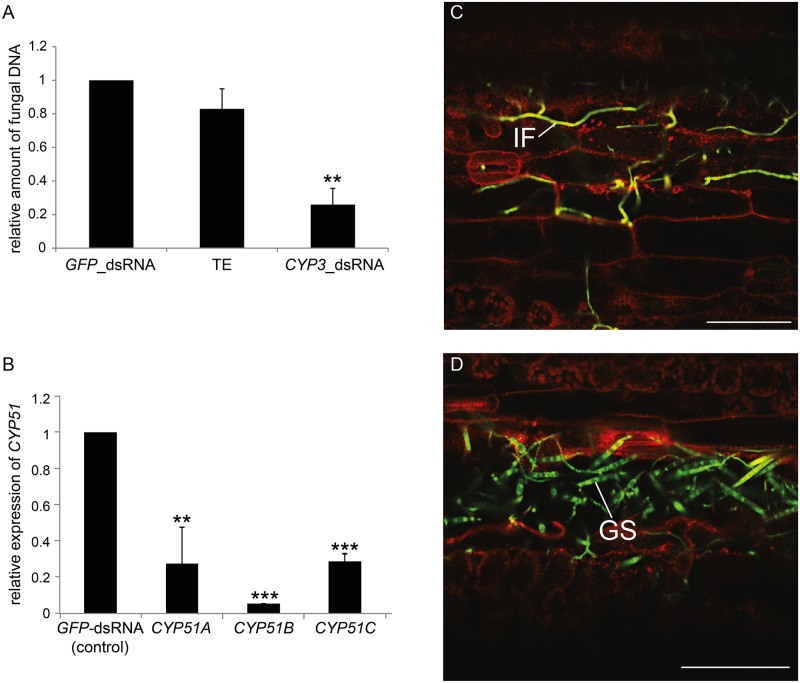
(A-D) SIGS-mediated semi-systemic control of *Fusarium graminearum*. **(A)** Upper parts of detached second leaves of three-week-old barley were sprayed evenly with *CYP3*-dsRNA, TE, and *GFP*-dsRNA, respectively. After 48 h, the non-inoculated, semi-systemic (distal) tissue was drop-inoculated with 2 × 10^4^ conidia mL^−1^ of Fg-IFA65_GFP_; the relative amount of fungal DNA in distal tissue, as measured by qPCR at 6 dpi, was reduced in *CYP3*-dsRNA-treated leaves. Bars represent mean values ± SDs of three independent experiments. The reduction of fungal growth on *CYP3*-dsRNA-sprayed leaves was statistically significant (**P < 0.01; Student´s t test). **(B)** Gene-specific qPCR analysis of *CYP51A*, *CYP51B*, and *CYP51C* transcripts at 6 dpi in distal leaf areas. Bars represent mean values ±SDs of three independent sample collections. The reduction in *CYP51* expression in leaves sprayed with *CYP3*-dsRNA compared with *GFP*-dsRNA-sprayed controls was statistically significant (**P < 0.01, ***P < 0.001; Student´s t test). **(C,D)** Microscopy of fungal growth at semi-systemic sites of drop-inoculation with Fg-IFA65_GFP_. **(C)** Successful fungal colonization (green) on TE-sprayed leaves. Profuse hyphal growth is seen inside the cells (plasma membrane stained with RH414 is highlighted in red) **(D)** Hyphal formation is strongly reduced and confined to the inoculated leaf area on *CYP3*-dsRNA-sprayed leaves. Impaired spore germination was observed in the area around the inoculation site while the surrounding cells remained free of colonization. (IF, infection hyphae; GS, germinating spore). Photographs for C and D were taken at 6 dpi.

Mobile cell non-autonomous inhibitory RNAs that spread gene silencing into adjacent cells and tissues have been observed in various plants [21–23]. Encouraged by the observed reduction in GFP fluorescence in Fg-IFA65_GFP_ upon infection of leaf segments that did not receive a direct *GFP*-dsRNA spray (see Fig 1), we tested whether locally sprayed *CYP3*-dsRNA confers gene silencing in Fusarium infecting distal, non-sprayed segments of barley leaves. To this end, the upper part of detached leaves (local tissue) was sprayed with 20 ng μL^-1^ *CYP3*-dsRNA, *GFP*-dsRNA, or TE, while the lower part (distal tissue) was covered by a plastic tray to prevent direct dsRNA contamination. After 48 h, the distal, non-sprayed part of the leaves was drop-inoculated with Fg-IFA65_GFP_; six days later, resistance to fungal infection was assessed. Distal leaf areas of *CYP3*-dsRNA-treated leaves developed substantially smaller lesions as compared to leaves sprayed with *GFP*-dsRNA or TE (S2 Fig) indicating that the silencing signal was basipetally transported. Consistent with this finding, the amount of fungal DNA as determined by qPCR was greatly reduced in the distal leaf area as compared to the control treatments (Fig 3A). The relative amounts of fungal *CYP51A*, *CYP51B* and *CYP51C* transcripts were strongly reduced on average by 72% (*CYP51A*), 90% (*CYP51B*), and 71% (*CYP51C*) as compared with control (*GFP*-dsRNA) treatment (Fig 3B). Confocal microscopy of fungal inoculation sites in distal leaf areas confirmed that, on TE-treated leaves, Fg-IFA65_GFP_ conidia had germinated and colonized tissue next to the inoculation site (Fig 3C). In contrast, fungal mycelia on *CYP3*-dsRNA-treated leaves were only visible at the inoculation sites, and the surrounding leaf tissue was free of infection hyphae (Fig 3D). Consistent with this, the large number of fungal conidia with very short germ tubes at the inoculation sites of *CYP3*-dsRNA-treated leaves indicated that fungal germination was strongly impaired. We also tested whether the silencing signal was transported in the acropetal direction. Segments were sprayed with 20 ng μL^-1^ *CYP3*-dsRNA and subsequently drop-inoculated in the distal leaf area. Fusarium infections were also reduced in the acropetal experimental set up (S3A Fig) as shown by macroscopic inspection (S3B Fig) and qPCR quantification of fungal DNA (S3C Fig).

### Uptake and processing of sprayed *CYP3*-dsRNA

**Fig 4:**
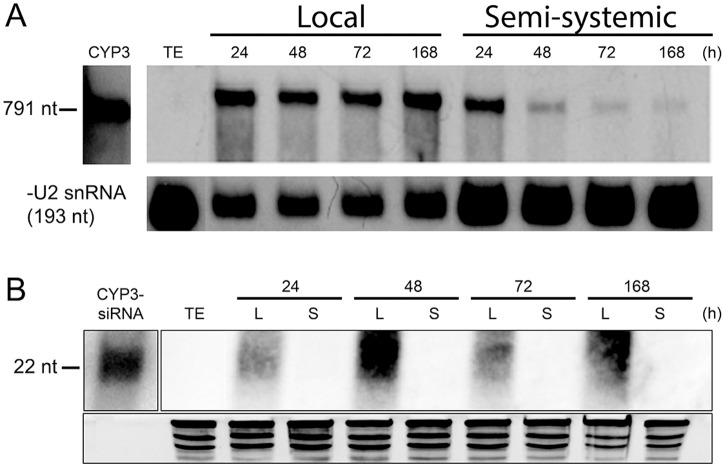
(A,B) Northern gel blot analysis of *CYP3*-dsRNA and *CYP3*-dsRNA-derived siRNA accumulation in local and distal (semi-systemic) barley leaf areas. **(A)** Detection of 791 nt long *CYP3*-dsRNA precursor in pooled leaf tissue from non-infected leaves using [α-32P]-dCTP labeled *CYP3*-dsRNA as probe. Local (L) and distal (semi-systemic [S]) leaf segments were sampled separately at the indicated times after spraying with *CYP3-*dsRNA. No signal was detected in samples from TE-sprayed plants. **(B)** Recording *CYP3*-dsRNA-derived small RNAs in local and distal (semi-systemic) leaf areas using [α-32P]-dCTP labeled *CYP3*-dsRNA as probe. In this experiment, small RNAs could not be detected in distal (non-sprayed) tissues. siRNA generated *in vitro* by a commercial Dicer preparation from *CYP3*-dsRNA was used as positive control. No signal was detected in samples from TE-sprayed plants. Ethidium bromide-stained rRNA served as the loading control. Signals originate from the same membrane but different exposure times.

To further explore the SIGS mechanism, we investigated whether the spray-applied long *CYP3*-dsRNA is translocated in the plant tissue and/or processed by the plant’s silencing machinery independent of fungal infections. Following *CYP3*-dsRNA treatment, local (sprayed) and distal (non-sprayed) leaf segments were harvested separately at 24, 48, 72, or 168 h after spraying. Northern blot analysis detected unprocessed 791 nt *CYP3*-dsRNA in both local and distal tissue (Fig 4A), showing that the long dsRNA is systemically translocated within the plant. In the local (sprayed) segment, *CYP3*-dsRNA was detected over the full time range, while it accumulated only transiently at early time points (24 h) after spraying in the distal (non-sprayed segments). This accumulation profile is consistent with the idea that the vast bulk of the *CYP3*-dsRNA fraction was absorbed via the cut surface of the detached leaf. Moreover, *CYP3*-dsRNA-derived 21 nt and 22 nt small interfering (si)RNAs also accumulated over the whole time range after spraying in the local leaf segments, demonstrating that *CYP3*-dsRNA was partly processed by the plant (Fig 4B). In this experiment, Northern analysis could not detect siRNAs in distal leaf parts, probably because the technique was not sensitive enough.

**Fig 5:**
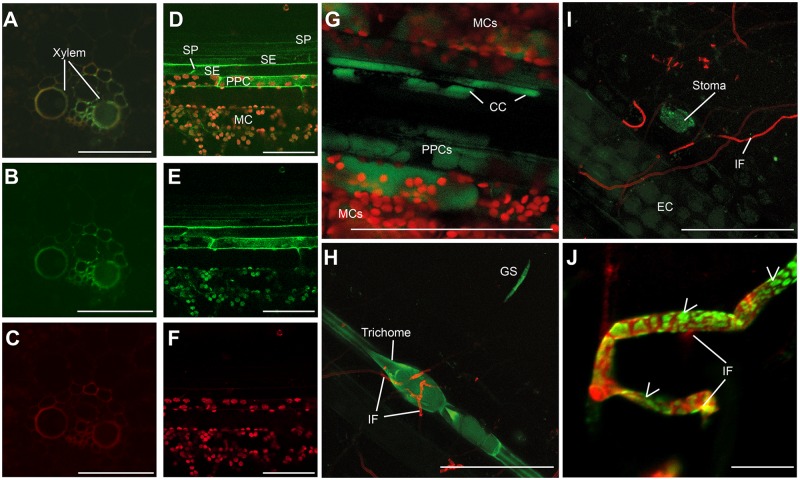
(A-J) Confocal laser scanning microscopy of ATTO 488-labeled *CYP3*-dsRNA_A488_ in locally sprayed barley leaves. **(A-C)** Detection of *CYP3*-dsRNA_A488_ (green) in xylem vessels of vascular bundles 24 h after spraying. **(D-G)** Longitudinal sections reveal uptake of *CYP3*-dsRNA_A488_ by cells of the phloem tissue at 24 h after spraying. SE, sieve element; CC, companion cell; SP, sieve plate; PPC, phloem parenchyma cell; MC, mesophyll cell. The red cells result from the autofluorescence of chloroplasts (F,G). **(H-J)** Leaf hair cells (trichome), stomata, germinating spores (GS) and fungal hyphae strongly accumulated *CYP3*-dsRNA_A488_. Fungal hyphae (IF) are stained with chitin-specific dye WGA-Alexa Fluor 594 (red) 24 h after inoculation. EC, epidermal cells. RNA signals in germinated conidia are marked by arrow heads. Scale bars 100 μm (A-H), 20 μm (F), and 10 μm (J).

To further investigate uptake and transport of sprayed *CYP3*-dsRNA, it was labeled with the green fluorescent dye ATTO 488 (*CYP3*-dsRNA_A488_) and sprayed onto barley leaves. The biological activity of *CYP3*-dsRNA_A488_ was indistinguishable from non-labeled *CYP3*-dsRNA as evidenced by reduced fungal infection and strong silencing of fungal *CYP51* genes upon spray application (S4A–S4C Fig). Moreover, using confocal laser scanning microscopy, a green fluorescent signal was detected in the vascular tissue at 24 hours after spraying leaves with 20 ng μl^-1^ *CYP3*-dsRNA_A488_. In leaf cross-sections, fluorescence was seen in the xylem (Fig 5A–5C). Inspection of longitudinal leaf sections revealed that the fluorescence was not confined to the apoplast but also was present in the symplast of phloem parenchyma cells, companion cells, and mesophyll cells, as well as in trichomes and stomata (Fig 5D–5I). When *CYP3*-dsRNA_A488_-sprayed leaves were inoculated with Fg-IFA65, the fluorescent signal also was detectable inside fungal conidia and germ tubes (Fig 5H) and fungal mycelium (Fig 5J and S5 Fig). Together these data show that *CYP3*-dsRNA is taken up by the plant and is transferred via the plant vascular system. Systemic translocation within the plant and accumulation by the fungus also raised the possibility that *CYP3*-dsRNA is processed by the fungus into inhibitory siRNAs to eventually target fungal *CYP51* genes.

**Fig 6:**
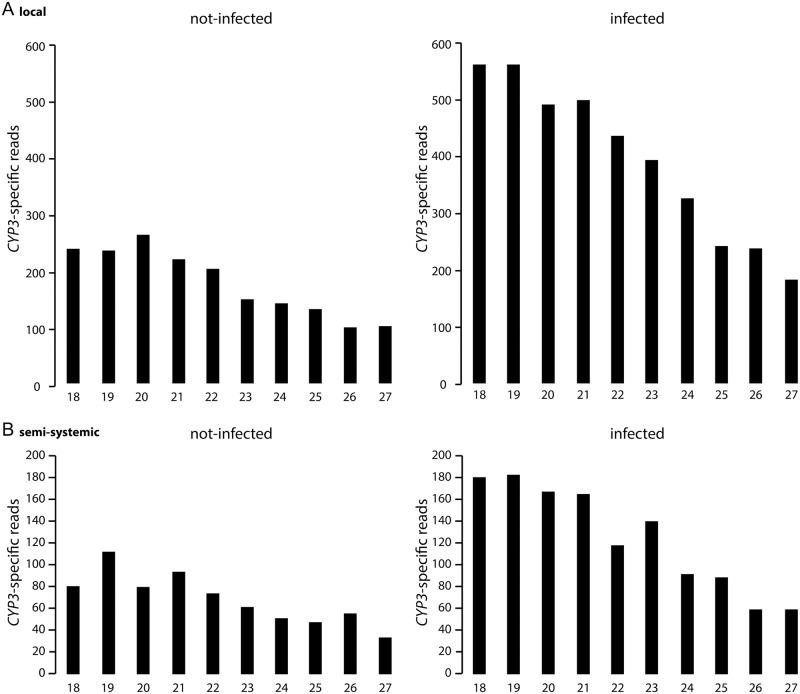
(A,B) RNA profiling (RNAseq analysis) of *CYP3*-dsRNA-derived RNAs in local and distal tissue of *CYP3*-dsRNA-treated barley leaves on Illumina HiSeq. Higher numbers of reads of *CYP3*-dsRNA-derived sRNAs were found in infected vs. non-infected leaves both in the sprayed (local [A]) and non-sprayed (semi-systemic [B]) leaf area at 6 dpi with strain Fg-IFA65.

To test this possibility, we first profiled *CYP3-*dsRNA-derived siRNAs in infected and non-infected leaves. Small RNA sequencing (RNAseq) analysis revealed distinctly different *CYP3*-dsRNA-derived siRNA profiles in mock- vs. Fg-IFA65-infected local and distal (non-sprayed) leaf segments (Fig 6A and 6B) with higher numbers of reads of *CYP3*-dsRNA-derived siRNAs in infected leaves, and highest numbers of reads in locally-inoculated vs. distally-inoculated leaves. These data suggest that *CYP3*-dsRNA also is processed by the fungus and that the fungal silencing machinery is involved in SIGS and reduced fungal infections. Detection by RNAseq of *CYP3-*dsRNA-derived siRNA in the distal (non-sprayed) part of leaves also supported our interpretation that northern analysis failed to detect low amounts of these siRNAs due to sensitivity problems.

### Fungal DICER-LIKE-1 is required for efficient SIGS in systemic leaf areas

**Fig 7:**
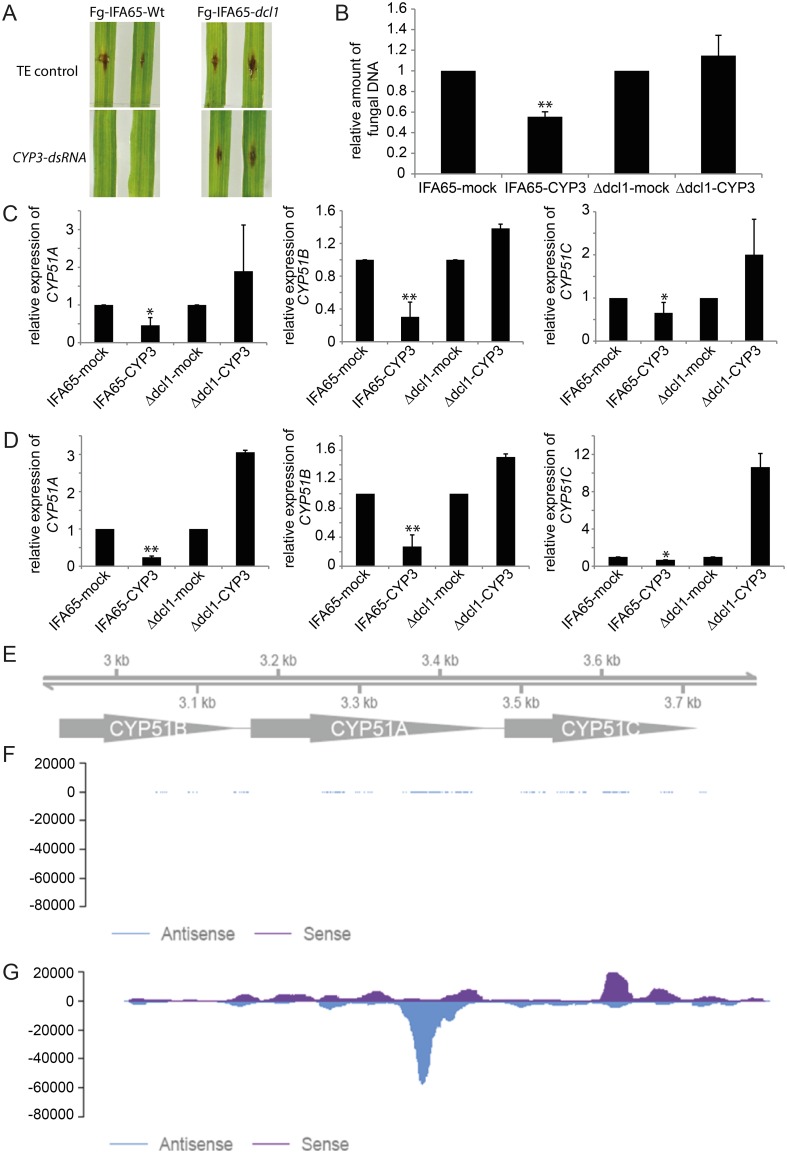
(A-E) The fungal silencing machinery is required for efficient SIGS in distal leaf parts. **(A,B)** The fungal *dicer-like-1* mutant Fg-IFA65_Δdcl-1_ heavily infected barley leaves despite a prior spray-treatment with *CYP3*-dsRNA. Photographs were taken at 6 dpi. **(C)** Gene-specific qPCR analysis of *CYP51A*, *CYP51B*, and *CYP51C* transcripts in the wild type Fg-IFA65 and the mutant Fg-IFA65_Δdcl-1_ at 6 dpi in the distal, semi-systemic leaf areas. **(D)** Inhibition of *CYP51* gene expression upon *CYP3*-dsRNA treatment of axenically grown Fg-IFA65_-_ Bars represent mean values ±SDs of three independent sample collections. The reduction in *CYP51* expression in samples treated with *CYP3*-dsRNA compared with mock-treated controls was statistically significant (*P < 0.05, **P < 0.01; Student´s t test). **(E-G)** Profiling of *CYP3*-dsRNA-derived sRNAs in axenically grown Fg-IFA65. (E) Scaffold of the 791 nt long *CYP3*-dsRNA. The fragments of *CYP51* genes are indicated. (F,G) Total sRNAs were isolated from axenically-cultured Fg-IFA65. sRNA reads of fungal sRNAs from untreated (F) and *CYP3*-dsRNA-treated (G) fungal cultures are mapped to the sequence of *CYP3*-dsRNA.

To further substantiate involvement of the fungal silencing machinery, we generated a fungal *dcl-1* mutant (Fg-IFA65_Δdcl-1_) that is deficient for DICER-LIKE 1 (S6 Fig), a critical component of the fungal silencing machinery that produces siRNA from long dsRNA stretches. Fg-IFA65_Δdcl-1_ and the wild type Fg-IFA65 were indistinguishably virulent on TE-sprayed barley leaves (Fig 7A), showing that fungal DCL-1 is not required for successful leaf infections. However, in contrast to Fg-IFA65, the mutant Fg-IFA65_Δdcl-1_ also heavily infected distal areas of *CYP3*-dsRNA-treated barley leaves (Fig 7B), suggesting that the mutant strain is not amenable to SIGS. We concluded that the fungal silencing machinery appears to be indispensable for *CYP3*-dsRNA-mediated SIGS at systemic areas in the barley-*Fusarium graminearum* pathosystem. To further confirm that FgDCL-1 is required for *CYP51* target gene silencing, levels of *CYP51A*, *CYP51B* and *CYP51C* transcripts were compared by qPCR in the wild type vs. the *dcl-1* mutant on infection of *CYP3*-dsRNA sprayed leaves. The relative amounts of transcripts were reduced in Fg-IFA65 on average by 50% (*CYP51A*), 70% (*CYP51B*), and 40% (*CYP51C*) as compared with TE (control) treatment. In contrast, expression of *CYP51* targets was not reduced in the Fg-IFA65_Δdcl-1_ mutant (Fig 7C).

We additionally conducted an *in vitro* experiment to further demonstrate the requirement of FgDCL-1 for *CYP3*-dsRNA-mediated silencing of fungal *CYP51* genes. Mycelia of axenic cultures of Fg-IFA65 and Fg-IFA65_Δdcl-1_ were treated with *CYP3*-dsRNA. Subsequently, expression of *CYP51* genes was recorded. Consistent with the leaf assay, the relative amounts of fungal *CYP51A*, *CYP51B* and *CYP51C* transcripts were reduced in the wild type Fg-IFA65 but not in the Fg-IFA65_Δdcl-1_ mutant (Fig 7D). Confirmatory total sRNAs profiling by RNAseq in axenically-grown Fg-IFA65 revealed a range of sRNAs originating from *CYP3*-dsRNA (Fig 7E–7G and S2 Table), further proving that the fungus can process *CYP3-*dsRNA. Suspiciously, the majority of siRNA species mapped to sites in the *CYP51A* gene fragment of the *CYP3-*dsRNA. Further work must show if this profile is a result of the physical structure of the dsRNA.

### *CYP3*-dsRNA-derived siRNAs also confer SIGS

The failure to detect *CYP3*-dsRNA-derived siRNA in the distal area of *CYP3*-dsRNA-sprayed leaves by northern analysis along with the compromised SIGS phenotype of the mutant Fg-IFA65Δdcl-1 suggested that the concentration of siRNA in the distal leaf parts was too low to mediate silencing of *CYP51* genes in the fungus. Alternatively, Fusarium is generally unable to absorb siRNA from barley leaves. To address these possibilities, we sprayed barley leaves with high concentration of *CYP3*-dsRNA-derived siRNAs (20 ng μl^-1^) and subsequently inoculated local (sprayed) and distal (non-sprayed) leaf segments with Fg-IFA65. We found that the fungus was strongly inhibited by these siRNAs both in the local (S7A and S7C Fig) and distal leaf segments (S7B and S7C Fig) as compared with a control (*GFP-*dsRNA-derived siRNAs). Consistent with this, *CYP3-*dsRNA-derived siRNA also reduced the expression of *CYP51* genes of the fungus growing on local (S7D Fig) and distal leaves segments (S7E Fig), which shows that *F*. *graminearum* also can ingest inhibitory siRNAs from plant tissue. In clear support of this notion and consistent with the finding that *CYP3*-dsRNA-derived siRNA accumulated to higher concentration in leaf areas directly sprayed with *CYP3*-dsRNA, mutant Fg-IFA65_Δdcl-1_ was not compromised in SIGS when drop-inoculated directly to the sprayed leaf area (S8 Fig).

### SIGS-mediated fungal inhibition is independent of innate immune responses

**Fig 8:**
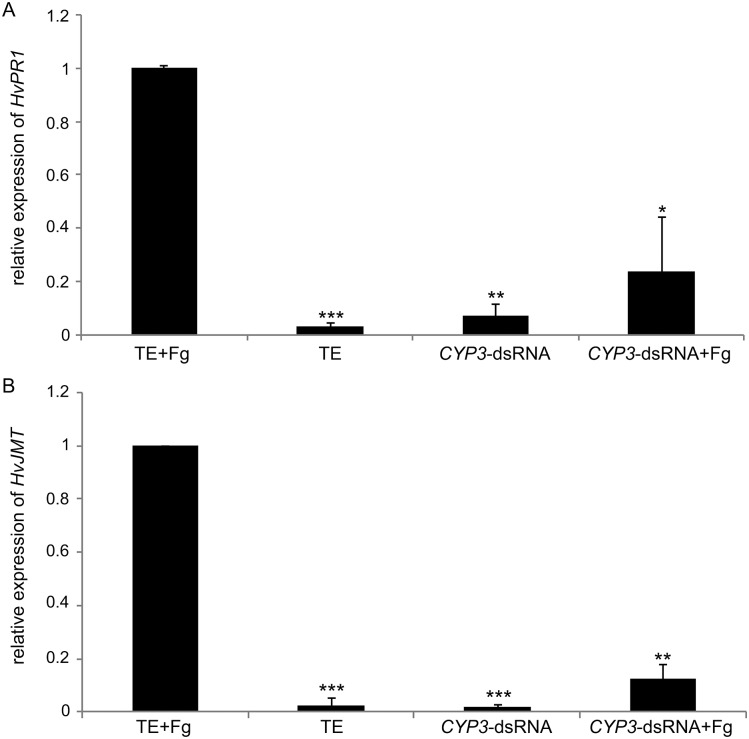
(A,B) Defense-related salicylate- and jasmonate-responsive genes are not induced by *CYP3*-dsRNA. Detached second leaves of three-week-old barley were sprayed with 20 ng μL^-1^ *CYP3*-dsRNA or TE (control), respectively, and 48 h later drop-inoculated with Fg-IFA65. Leaves were harvested 6 dpi and analyzed for gene expression by qPCR: **(A)** *Pathogenesis-related* 1 (*HvPR1*) and **(B)** *S-adenosyl-l-methionine*:*jasmonic acid carboxyl methyltransferase* (*HvJMT*). Both genes are highly responsive to Fg-IFA65 but not to *CYP3*-dsRNA or TE treatment. Please note that a combined treatment of *CYP3*-dsRNA followed by Fg-IFA65 48 h later also did not induce these marker genes, which shows independently that fungal development on *CYP3*-dsRNA-treated leaves is strongly inhibited.

In mammalian cells, perception of certain dsRNAs via toll-like receptors triggers an inflammation response [24,25]. Therefore, we assessed whether *CYP3*-dsRNA elicits an innate immune response called pattern-triggered immunity (PTI) [26], when sprayed onto barley leaf segments. To this end, expression of barley genes that are indicative of the canonical defense-related salicylate- and jasmonate-dependent pathways [27] was evaluated. Expression of salicylate-responsive *pathogenesis-related 1* (*HvPR1*) and jasmonate-responsive S*-adenosyl-l-methionine*:*jasmonic acid carboxyl methyltransferase* (*HvJMT*) in TE-treated leaves was strongly induced by Fg-IFA65, but not by *CYP3*-dsRNA treatment (Fig 8). Furthermore, Fg-IFA65-induced expression of either gene was much lower in *CYP3*-dsRNA-treated leaves as compared with TE-treated leaves. This result strongly suggests that *CYP3*-dsRNA does not induce PTI in barley, and that the SIGS mechanism does not rely on activation of canonical defense pathways. The finding also is relevant when considering the fitness cost, and thus yield, of the SIGS strategy.

## Discussion

In this study, we show that delivery of long noncoding double-stranded RNA targeting the three *CYP51* genes of the necrotrophic ascomycete fungus *Fusarium graminearum* via spray application effectively reduces the development of the pathogen on barley leaves. Thus, our work further supports the idea that RNA could be used as a chemical treatment to control plant diseases.

Next to the economic and ecologic consideration about the deployment of antimicrobial RNAs as a new plant protection measure, elucidating the molecular mechanisms of RNA-based disease control is a key for successful future implementation. While plant-derived transgene-mediated silencing of target genes in plant pathogens and pests (a mechanism known as host-induced gene silencing [HIGS]) has been frequently reported [12,14,19], few examples have demonstrated the efficiency of exogenous RNA delivery to kill plant attackers. HIGS virtually is based on the plant’s silencing machinery, whereas the mechanism of gene silencing by exogenously delivered dsRNA constitutes a more complex situation. For instance, diverging questions are possible involvement of the silencing machineries of host and/or the pathogen/pest, the requirement of local and remote transport of channeled dsRNA molecules, and the problem of dsRNA transport at the apoplast–symplast interface.

Using the *F*. *graminearum*—barley pathosystem as a model to study the mechanism of exogenously applied inhibitory dsRNA was motivated by the fact that Fusarium Head Blight and Crown Rot cause serious problems worldwide including food and feed safety issues due to mycotoxin contamination of cereals and maize. Focusing on fungal *CYP51* genes as targets for silencing was reasonable because our previous work provided proof-of-concept that transgene-mediated silencing of these genes effectively reduced fungal development in Arabidopsis and barley. More than that, direct treatment of *F*. *graminearum* with inhibitory dsRNA matching *CYP51* gene sequences had been demonstrated to inhibit fungal development in axenic cultures [19].

The previous finding of impaired fungal growth in axenic cultures, when treated with a 791 nt dsRNA (*CYP3*-dsRNA) targeting the three fungal genes *CYP51A*, *CYP51B*, and *CYP51C*, let us speculate that the fungus can process *CYP3*-dsRNA into siRNA that interfere with the expression of *CYP51* genes. Gene annotation of *F*. *graminearum*’s genome (http://www.broadinstitute.org) predicted genes coding for two ARGONAUTE-like proteins, two DICER-like proteins and five RNA-dependent RNA Polymerases (RDR), suggesting that the RNAi pathway is functional [28]. Consistent with these findings, RNAseq analysis of axenically grown *F*. *graminearum*, treated with *CYP3*-dsRNA, showed high numbers of reads of *CYP3*-dsRNA-derived siRNA (Fig 7). Together these data showed that *F*. *graminearum* possesses a functional gene silencing system, which is a prerequisite for disease control by SIGS.

To test the antifungal activity of *CYP3*-dsRNA and their siRNA derivatives, we used a detached leaf assay that enabled us to assess fungal growth in local (directly sprayed) and distal (semi-systemic, non-sprayed) leaf segments. Using this approach, we could demonstrate that inhibitory dsRNA translocated via the plant vascular system and eventually was absorbed by the pathogen from leaf tissue (Fig 5). The profile of inhibitory dsRNA accumulation, as demonstrated by northern blot analysis (Fig 4) and RNAseq (Fig 6), showed that both long *CYP3-*dsRNA and plant-processed *CYP3*-dsRNA-derived siRNA accumulate in the plant vascular system, though translocation of siRNA seems to be less efficient and thus siRNA concentration at the remote infection sites probably was not high enough to induced SIGS. Consistent with this notion, *in planta* produced *CYP3*-dsRNA-derived siRNAs was detected in the distal leaf parts only by the more sensitive RNAseq technique but not by northern analysis. Nevertheless, spraying high concentrations of *CYP3*-dsRNA-derived siRNA (20 ng μL^-1^) induced the SIGS process (S7 Fig), demonstrating that the fungus is able to absorb siRNAs from barley leaves.

Because of the less efficient movement of siRNAs, transport and translocation of the unprocessed 791 nt dsRNA has a critical role in the SIGS process, demonstrated by a compromised SIGS phenotype of the Fusarium mutant Fg-IFA65_Δdcl-1_ at distal leaf segments (Fig 7) but not at local, directly sprayed leaf segments (S8 Fig). Compromised DICER activity resulted in the fungus inability to cleave *CYP3*-dsRNA into siRNA, thus interrupting the RNA interference mechanism in case the concentration of *CYP3*-dsRNA-derived siRNA is not sufficiently high. Our finding that the unprocessed long dsRNA could be absorbed from leaf tissue has further implications for future disease control strategies using dsRNA. There are good arguments that application of longer dsRNAs might be more efficient than application of siRNAs given there more efficient translocation. Long dsRNA would be processed into many different inhibitory siRNA by the fungus, which not least also could be an issue when considering the risk of compound resistance emerging in a pathogen under field test conditions. Thus, based on our findings, further research is required to establish rules for optimal dsRNA structures, considering e.g. molecule lengths, combinatorial order of gene fragments, target sites in a given gene target, and the number of genes targeted by one dsRNA. Supporting the requirement for more information as to the design of dsRNA probes, RNAseq analysis revealed that most of the *CYP3*-dsRNA-derived siRNAs, accumulating in the axenic fungal mycelium, were not equally distributed at the *CYP3-*dsRNA scaffold, but accumulated at the *CYP51A* gene fragment (Fig 7 and S2 Table). Further analysis is required to explain this bias in the production of siRNAs from *CYP3*-dsRNA.

Our results are also consistent with the view that inhibitory dsRNA is more effectively absorbed by the fungus through infection hyphae that have intimate contact to plant tissue (compare *CYP51* gene expression in Figs 2 and 3). How these hyphae differ from the fungal germ tubes and extracellular hyphae is however yet unclear although distinct biochemical modifications of fungal hyphae that penetrate host plants and are involved in nutrient uptake have been demonstrated [29,30]. Thus it is likely that these specialized, leaf tissue colonizing hyphae show dsRNA uptake that is superior over germ tubes.

Our data are consistent with reports showing that silencing signals in plants are mobile [31,32]. The design of our experiments based on the previous finding that sRNAs, just as viroids [33], preferably move via the vascular system in the source-to-sink direction although some reports discussed transport in the opposite route (for review see [21]). Source-to-sink movement is one reason why the phloem rather than the xylem is generally considered as the conduit for movement of the silencing signal. This hypothesis is supported by the finding that the xylem sap, which transports water and ions, commonly is free of RNA [34]. However, spray application of dsRNA onto detached leaves cannot be compared with the situation in an intact leaf. Exogenously applied dsRNA first reached the apoplast, including the xylem (Fig 5), and subsequently translocated into the symplast by a yet unknown mechanism. Consistent with this, we could also demonstrate acropetal movement of the silencing signal that resulted in the inhibition of the fungus in distal not sprayed leaf areas (S3 Fig). Apoplastic movement of RNA has been proposed, e.g. to explain how maternally expressed siRNAs could be transferred from the endosperm of developing seeds into the symplastically isolated embryo [35].

Regardless of how target-specific inhibitory RNAs are applied–by transgene delivery (HIGS) or spray (SIGS)–the use of target-specific inhibitory RNAs to confer plant protection potentially is an alternative to conventional chemicals because they are i) highly specific and solely depending on their nucleotide sequence and ii) can be developed against an unlimited range of pathogens provided that the RNAi machinery is in place. Given the accumulation of dsRNA in the plant phloem (Fig 5), sucking insects also are realistic SIGS targets as their efficient control by HIGS has been largely demonstrated [36,37]. Certainly, many questions have to be addressed in the future to eventually judge the agronomical potential of SIGS, including the costs of RNA applications and their stability under field conditions. Hence, more research is required to develop application strategies, including improved uptake by compound design and use of chemical formulations. Another yet unassessed issue is the risk that microbial strains become insensitive to a commercial dsRNA product. Such scenario could probably be resolved by application of dsRNA mixtures that target different regions in one gene or even different genes. Moreover, a commercial dsRNA product should be designed not to have off-target effects in other organisms that might be relevant in the respective agroecosystem, including beneficial fungi and bacteria. In this respect, it is important to understand that both plants [12] and fungi [38] support the production of secondary siRNAs, meaning there is a potential for transitivity and amplification. It is therefore possible that low abundance inhibitory dsRNA sprayed onto the plant triggered a large silencing effect in Fusarium via these secondary RNAs. Importantly, when considering the regulatory issue of RNA-based plant protection it is crucial to emphasize that the principles of SIGS and HIGS rely on the mechanisms found for trans-kingdom communication in mutualistic and parasitic interactions, and thus is a natural phenomenon [12,13,14].

Apart from the dsRNAs prospects in future plant protection strategies, there is an additional technological potential in developing new pesticides. The simple phenotyping adopted by the SIGS screens renders them a powerful tool for genetic studies to assess compound targets with high efficiency and low costs. Thus, the present data provide essential scientific information on a fundamentally new plant protection strategy, thereby opening novel avenues for improving crop yields in an environmentally friendly and sustainable manner.

## Materials and Methods

### Plant and fungal materials

The spring barley (*Hordeum vulgare*) cultivar (cv.) Golden Promise was grown in a climate chamber under 16 h light photoperiod (240 μmol m^-2^ s^-1^ photon flux density) at 18°C/14°C (day/night) and 65% relative humidity. The wild type *Fusarium graminearum* strain Fg-IFA65 was described earlier [20]. Plates were incubated at room temperature under constant illumination from one near-UV tube (Phillips TLD 36 W/08) and one white-light tube (Phillips TLD 36 W/830HF). Fungal strains were cultured on synthetic nutrient-poor agar (SNA)-medium [39].

Generation of transgenic *F*. *graminearum* (Fg-IFA65_GFP_), expressing a jellyfish *green fluorescence protein* (*GFP*) gene under the *Neurospora crassa isocitrate lyase* gene promoter (PCII) [40], was performed as described [41].

For generation of the *DICER LIKE-1* Fg-IFA65_Δdcl-1_ knock-out mutant, a homologous recombination strategy was used. The two homologous recombination segments USS and DSS (~1 kb each), representing promoter and termination regions of the *FgDCL-1* gene, were selected based on the sequence information available at the *Fusarium graminearum* genome database (http://www.broadinstitute.org), and were PCR amplified. Primers used for USS (DCL_1_USS_KpnI_F and DCL_1_USS_KpnI_R), and DSS (DCL_1_DSS_HindIII_F and DCL_1_DSS_HindIII_R) are listed in S1 Table. The USS and DSS were cloned into flanking sites of the *hph* cassette of the pPK2 binary vector [38] using USER enzyme mix (New England Biolab, Inc., Ipswich, MA, USA) in *Escherichia coli*. The resultant plasmid was confirmed for proper orientation of cloned inserts in the vector by PCR conducted using USS/DSS and vector-specific primers and then by sequencing the PCR products.

The pPK2::Δ*Fgdcl-1* binary plasmid containing two *Fgdcl-1* USS and DSS was transformed into *Agrobacterium tumefaciens* strain LBA4404 by electroporation, and transformants were analyzed by conducting restriction analysis. The ATMT of *F*. *graminearum* was based on a modified protocol [42]. Briefly, *A*. *tumefaciens* LBA4404 containing the pPK2::Δ*Fgdcl-1* plasmid was grown overnight in LB medium at 28°C (25 μg mL^-1^ kanamycin, 25 μg mL^-1^ rifampicin, and 5 μg mL^-1^ tetracycline). The next day, 10 ml of LB medium supplemented with above mentioned antibiotics and 200 μM acetosyringone was inoculated with 100 μl of the *A*. *tumefaciens* culture. This *A*. *tumefaciens* cell suspension with an OD_600_ of 0.5 to 0.7 was mixed with *F*. *graminearum* conidial suspensions (10^5^−10^6^ mL^-1^) in liquid SNA medium in equal proportions [1:1(v/v)]. Aliquots of 200 μl of the mixture were spread on black filter paper circles (Grade 551; Whatman Inc., Piscataway, NJ, USA), which were overlaid on SNA plates containing 200 μM acetosyringone and incubated for 2 days in the dark at RT until mycelial growth was observed on the filter paper. Transformants were selected on SNA medium supplemented with 50 μg mL^-1^ of hygromycin B (Sigma, St. Louis, MO, USA) and 300 μg mL^-1^ ticarcillin (Fisher Scientific, Pittsburgh, PA, USA).

### dsRNA synthesis

The stacked clone (*CYP51 B-A-C*) [19] covering sequences of the three cytochrome P450 lanosterol C-14α-demethylase genes *CYP51A* (FGSG_04092), *CYP51B* (FGSG_01000), and *CYP51C* (FGSG_11024) from *F*. *graminearum* was used as template for the synthesis of a 791 nt long *CYP3-*dsRNA [19]. The pLH6000-Ubi::sGFP plasmid [43] was used as template for the synthesis of a 720 nt long *GFP*-dsRNA (S1 Fig). dsRNA was generated using MEGAscript RNAi Kit (Invitrogen) following MEGAscript protocols. Primer pairs T7_F and T7_R with T7 promoter sequence at the 5`end of both forward and reverse primers were designed for amplification of dsRNA (S1 Table).

### sRNAs synthesis

sRNAs were generated using PowerCut Dicer (Thermo Scientific). Following the PowerCut Dicer protocol, *CYP3-*dsRNA or *GFP*-dsRNA was used as template for Dicer cleavage.

### Spray application

Detached leaf assay: Detached leaves of three-week-old barley plants were transferred into square Petri dishes (120 x 120 x 17mm) containing 1% agar. For spray application, dsRNA was diluted in 500 μL water to a final concentration of 20 ng μL^-1^, corresponding to 10 μg dsRNA per plate. For sRNA application, the reaction mixture of DICER-cleaved dsRNA was used at a final concentration of 10 μg siRNA diluted in 500 μL^-1^ water per plate. Leaves were sprayed using a spray flask (10 mL capacity). Each dish containing six detached leaves was evenly sprayed. For the semi-systemic setup, detached leaves were covered before spraying with a plastic tray leaving only the upper part (approximately 1 cm) uncovered. After spraying, dishes were kept open until the surface of each leaf was dried (approximately 2 h). After an indicated lag time, leaves were drop-inoculated with 20 μL of 2 × 10^4^ fungal conidia mL^−1^. Closed dishes were incubated for 6 d at approximately 20°C on the lab bench. Alternatively, one-week-old barley seedlings were sprayed in the first leaf stage with 20 ng μL^-1^ dsRNA, and spray-inoculated three weeks later with 2 × 10^4^ conidia mL^−1^ of Fg-IFA65. Inoculated plants were grown for three weeks in a growth chamber before evaluating the infection symptoms.

### *In vitro* axenic cultures

Fg-IFA65 and Fg-IFA65_Δdcl-1_ were cultured on synthetic nutrient SNA-medium. Plates were incubated at room temperature under constant illumination from one near-UV tube (Phillips TLD 36 W/08) and one white-light tube (Phillips TLD 36 W/830HF). Conidia were harvested from one-weak-old cultures with a sterile glass rod and sterile water [19]. *CYP3*-dsRNA was added to the fungal samples. Plates were incubated at room temperature. Gene expression studies were performed 24 h post *CYP3*-dsRNA treatment.

### Quantification of fungal infection and transcript analysis

The relative amount of fungal DNA was measured using qPCR to quantify fungal infection. DNA was extracted using the CTAB method [44]. Expression analysis of the three fungal *CYP51* genes as well as plant defense marker genes *PR1* and *JMT*, respectively, was performed using qPCR. RNA extraction from infected leaves was performed with TRIzol (Invitrogen) following the manufacturer’s instructions. Freshly extracted mRNA was used for cDNA synthesis using QuantiTect Reverse-Transcription kit (Qiagen). For qPCR 10 ng of cDNA was used as template in the Applied Biosystems 7500 FAST realtime PCR system. Amplifications were performed in 7.5 μL of SYBER green JumpStart Taq ReadyMix (Sigma-Aldrich) with 0.5 pmol oligonucleotides. Each sample had three repetitions.

To quantify the amount of fungal DNA, primers were used for assessing expression of the fungal β-tubulin gene (FGSG_09530) with reference to barley ubiquitin gene (S1 Table). Primers were used for assessing expression of target *CYP51* genes with reference to β-tubulin gene (S1 Table). After an initial activation step at 95°C for 5 min, 40 cycles (95°C for 30 sec, 57°C for 30 sec, 72°C for 30 sec) were performed. Ct values were determined with the 7500 Fast software supplied with the instrument. Transcript levels of β -tubulin gene were determined via the 2-Δ Δ Ct method [45] by normalizing the amount of target transcript to the amount of reference transcript.

### Small RNA library production and sequence analysis

RNA enriched for the sRNA fraction was purified from plant and fungal samples using the mirVana miRNA Isolation Kit (Life Technologies). Indexed sRNA libraries were constructed from these enriched sRNA fractions with the NEBNext Multiplex Small RNA Library Prep Set for Illumina (New England Biolabs) according to the manufacturer’s instructions. Indexed sRNA libraries were pooled and sequenced on the Illumina HiSeq and NextSeq 500 platforms and the sequences sorted into individual datasets based on the unique indices of each sRNA library. The adapters and indices were trimmed using Trimmomatic [46] version 0.2.2 and the reads were mapped to the *CYP3*-dsRNA vector sequence using bowtie2 [47] version 2.1.0. to identify sRNAs with a perfect match. Each library contained at least 5 million total reads.

### Northern blot analysis

For Northern blot analysis, 8 ng of total RNA from local region and 80 ng of systemic region or negative control (TE-mock) and 10 pg of *in vitro* transcribed *CYP3-*dsRNA was loaded onto a 6% denaturing polyacrylamide gel with DNA Molecular Weight Marker VIII (Roche), transferred to a nylon membrane. *CYP3-*dsRNA and U2 snRNA were detected using the DIG Labeling and Detection System (Roche) following the manufacturer’s instructions. Chemiluminescence was detected using X-ray films.

The *CYP3-*dsRNA probe was created by PCR with *CYP3-*dsRNA forward and reverse primer (S1 Table) on the stacked clone (*CYP51 B-A-C*) [19] using PCR DIG Labeling Mix (Roche). U2 snRNA loading control was amplified from cDNA created from total RNA using qScript Flex cDNA Kit (Quanta BioSciences) and primers U2 forward (TACCTTTCTCGGCCTTTTGG and U2 reverse (CAGCAGCAAGCTACTGTGGT). Gel purified probes were hybridized in NorthernMax Prehybridization/Hybridization Buffer (Ambion) at 45°C over night.

Northern blots for the detection of *CYP3*-dsRNA-derived siRNA were performed as described [19] using a 791 nt [α-32P]-dCTP labeled *CYP3*-dsRNA as probe.

### Confocal microscopy of fluorophore distribution

Twenty-four h after spraying fluorescing dsRNA were imaged using a Leica TCS SP2 (Leica Microsystems, Wetzlar, Germany) equipped with a 75-mW argon/krypton laser (Omnichrome, Chino, CA) and a water immersion objective (HCX APO L40x0.80 W U-V-l objective). Fluorescing dsRNA were imaged using a LSM 880 (Zeiss Microscopy GmbH, Jena, Germany) with the 488 nm laser line of an argon multiline laser (11.5 mW) and a HeNe 594 nm (1.3 mW) laser. Images were taken with a 20x objective (Plan-Apochromat 20x/0.8). Lambda stacks were created using the 32 channel GaAsP detector. Reference spectra with each pure fluorescence dye were recorded. The sample was inspected in Online Fingerprinting mode. Specific areas of the sample were imaged in lambda mode followed by Linear Unmixing with ZEN software (Zeiss, Jena, Germany). Fluorescent labeling of the dsRNA was performed using the Atto 488 RNA Labeling Kit (Jena Bioscience, Jena, Germany) following the manufacturer’s instructions. Leaves were sprayed with the labeled dsRNA and 24 h later drop-inoculated with 2 × 10^4^ Fg-IFA65 conidia mL^−1^. To assess whether dsRNA has an effect on fungal morphology, leaves were inoculated with Fg-IFA65_GFP_ and infected leaves were analyzed at 6 dpi.

For observation of phloem tissue, cortical cell layers were removed down to the phloem from the lower side of the main vein of a mature leaf. The leaf surface and longitudinal- as well as cross sections were stained with 4.3 μM of the membrane dye RH-414 (-N-(3-triethylammoniumpropyl)-4-(4-(4-(diethylamino)phenyl)butadienyl)pyridiniumdibromid) and/or with 5 μg mL^-1^ of the fungal hyphae dye wheat germ agglutinin (WGA) Alexa Fluor^®^ 594 conjugate (Invitrogen) for at least 10 min.

RH-414, WGA Alexa Fluor 594, and the autofluorescence of cell walls and chloroplasts were excited by the 564-nm line of the argon/krypton laser, while GFP and ATTO 488 were excited with the 488-nm line. For observation at the 590 nm and 510 nm wave lengths, respectively, a long pass filter was used. Digital images were processed with Adobe Photoshop to optimize brightness, contrast, and color and to enable an overlay of the photomicrographs.

### Statistical analysis

Analyses were performed in SigmaPlot 12 (Systat Software) using Student´s t-tests after data were tested for normality distribution (Shapiro-Wilk test).

### Genes mentioned in the text

*FgCYP51A* (FGSG_04092); *FgCYP51B* (FGSG_01000); *FgCYP51C* (FGSG_11024); *FgDCL1* (FGSG_09025); *ß-tubulin* (FGSG_09530); *HvPR1* (X74940); *HvJMT* (BAD33074.1); *HvUBQ* (M60175)

## Supporting Information

S1 Fig Partial DNA sequence of jellyfish *green fluorescent protein* (*GFP*) from which *GFP*-dsRNA is derived (forward strand). (TIF)

S2 Fig SIGS-mediated control of *F*. *graminearum* on leaves sprayed with *CYP3*-dsRNA (basipetal direction). Detached second leaves of three-week-old barley were locally sprayed with 20 ng μL^-1^ *CYP3*-dsRNA, TE (mock control), and *GFP*-dsRNA (negative control), respectively. After 48 h, leaves were drop-inoculated at the non-sprayed distal area (systemic; basipetal direction) with 2 × 10^4^ conidia mL^−1^ of Fg-IFA65 and evaluated for necrotic lesions at 6 dpi.
(TIF)

S3 Fig SIGS-mediated systemic control of *Fusarium graminearum* (acropetal direction). **(A)** Lower parts of detached second leaves of three-week-old barley were sprayed evenly with *CYP3*-dsRNA and TE, respectively. After 48 h, the non-sprayed, distal (acropetal direction) tissue was drop inoculated with 2 × 10^4^ conidia mL^−1^ of Fg-IFA65_GFP_. **(B)** Macroscopy of fungal growth at distal sites of drop-inoculation with Fg-IFA65_GFP_. Stronger fungal colonization was seen on TE-sprayed leaves. Photographs were taken at 6 dpi. **(C)** The relative amount of fungal DNA in distal tissue as measured by qPCR at 6 dpi, was reduced in *CYP3*-dsRNA-treated leaves. Bars represent mean values ± SDs of two independent experiments. The reduction of fungal growth on *CYP3*-dsRNA-sprayed leaves was statistically significant (*P < 0.05; Student´s t test).
(TIF)

S4 Fig Biological activity of fluorescent ATTO 488-labeled *CYP3*-dsRNA_A488_ on Fusarium infections in the distal, semi-systemic (non-sprayed) tissue of barley second leaves at 6 dpi. **(A)** *F*. *graminearum* infections are reduced in the distal tissue of leaves that received a 48 h pretreatment with *CYP3*-dsRNA_A488_ as compared to *GFP*-dsRNA_A488_ (control). **(B)** Quantification of fungal infection by qPCR. **(C)** Gene-specific quantification of *CYP51* transcripts by qPCR.
(TIF)

S5 Fig Confocal laser scanning microscopy of ATTO 488-labeled *CYP3*-dsRNA_A488_ in locally sprayed barley leaves. **(A)** Bright field microscopy of a fungal hyphae. **(B)** Hyphae strongly accumulated *CYP3*-dsRNA_A488_. **(C)** Hyphae stained with chitin-specific dye WGA-Alexa Fluor 594 (red). **(D)** Merge of B and C. RNA signals in germinated conidia are marked by arrow heads. Fungal infection hyphae (IF). Scale bars 10 μm.
(TIF)

S6 Fig Comparative qPCR analysis of the expression of *DICER-LIKE 1* in *Fusarium graminearum* wild type Fg-IFA65 and the *dcl-1* knock-out mutant Fg-IFA65_Δdcl-1_. (TIF)

S7 Fig *CYP3*-dsRNA-derived siRNAs are inducers of SIGS. **(A,B)** Detached second leaves of three-week-old barley were sprayed with 20 ng μL^-1^ of *CYP3*-dsRNA-derived sRNA (*CYP3*-siRNA) or *GFP*-dsRNA-derived sRNA (*GFP*-siRNA, control) (see online methods), and 48 h later drop-inoculated with Fg-IFA65. Infection symptoms were evaluated at 6 dpi in the local (sprayed) (A) and distal, semi-systemic (non-sprayed) tissue (B). **(C)** qPCR quantification of fungal DNA in local and distal leaf tissues after spray-application of *CYP3*-siRNA or *GFP*-dsRNA at 6 dpi. **(D,E)** Gene-specific quantification of *CYP51* transcripts by qPCR in local (D) and distal tissue (E) at 6 dpi. Bars represent mean values ± SDs of two independent experiments.
(TIF)

S8 Fig Fg-IFA65_Δdcl-1_ is not compromised in SIGS when inoculated directly to the sprayed leaf area. **(A)** Experimental design: Fusarium was drop-inoculated to the sprayed leaf area. **(B)** Both the fungal *dicer-like-1* mutant Fg-IFA65_Δdcl-1_ and the wt strain (Fg-IFA65) were inhibited by *CYP3*-dsRNA as compared to TE treatment. Photographs were taken at 6 dpi. **(C)** Quantification of fungal DNA by qPCR analysis confirmed the macroscopic analysis. Bars represent mean values ±SDs of two independent sample collections. The reduction of fungal growth in samples treated with *CYP3*-dsRNA compared with mock-treated TE controls was statistically significant (*P < 0.05, **P < 0.01; Student´s t test).
(TIF)

S1 Table Primers used in this study. (DOCX)

S2 Table sRNAs mapped to the CYP3-dsRNA sequence. (XLSX)
