# Supplementary figures and images for: An RNAi-Based Control of Fusarium graminearum Infections Through Spraying of Long dsRNAs Involves a Plant Passage and Is Controlled by the Fungal Silencing Machinery
Source: PLoS Pathog. 2016 Oct 13;12(10):e1005901. doi: 10.1371/journal.ppat.1005901 (PMC5063301; doi:10.1371/journal.ppat.1005901)

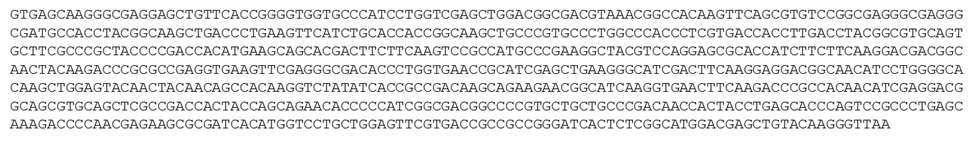

Supplement: S1 Fig — (TIF) [file ppat.1005901.s001.tif]

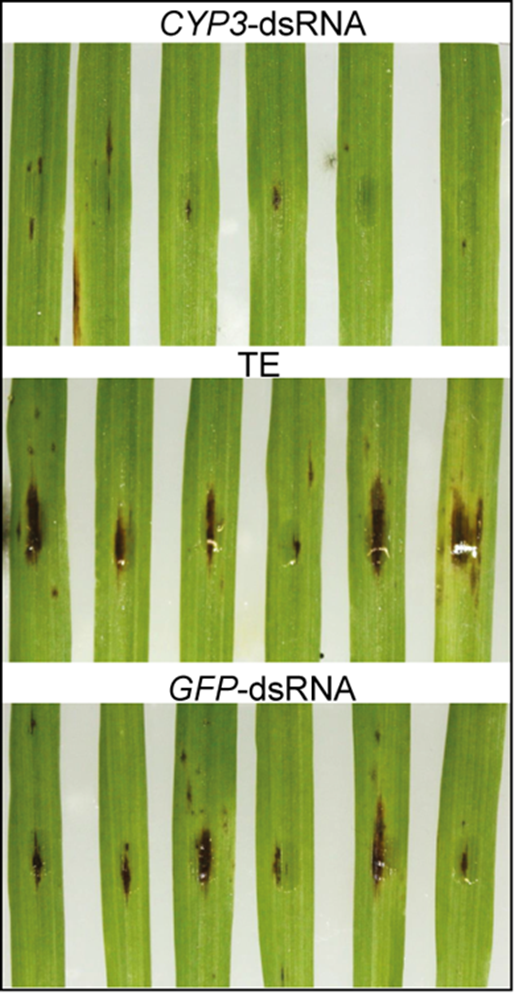

Supplement: S2 Fig — Detached second leaves of three-week-old barley were locally sprayed with 20 ng μL-1 CYP3-dsRNA, TE (mock control), and GFP-dsRNA (negative control), respectively. After 48 h, leaves were drop-inoculated at the non-sprayed distal area (systemic; basipetal direction) with 2 × 10^4^ conidia mL−1 of Fg-IFA65 and evaluated for necrotic lesions at 6 dpi. (TIF) [file ppat.1005901.s002.tif]

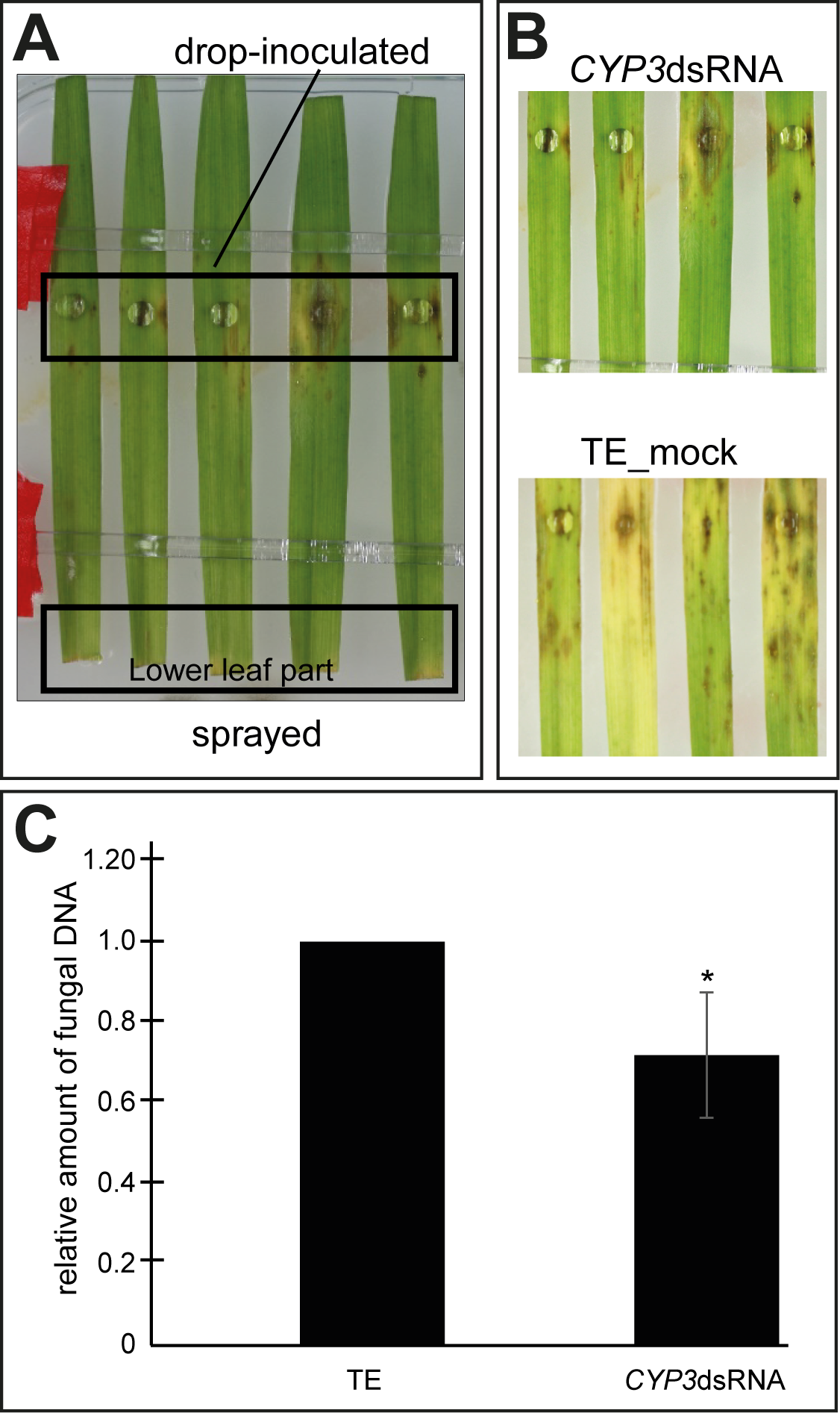

Supplement: S3 Fig — (A) Lower parts of detached second leaves of three-week-old barley were sprayed evenly with CYP3-dsRNA and TE, respectively. After 48 h, the non-sprayed, distal (acropetal direction) tissue was drop inoculated with 2 × 10^4^ conidia mL−1 of Fg-IFA65GFP. (B) Macroscopy of fungal growth at distal sites of drop-inoculation with Fg-IFA65GFP. Stronger fungal colonization was seen on TE-sprayed leaves. Photographs were taken at 6 dpi. (C) The relative amount of fungal DNA in distal tissue as measured by qPCR at 6 dpi, was reduced in CYP3-dsRNA-treated leaves. Bars represent mean values ± SDs of two independent experiments. The reduction of fungal growth on CYP3-dsRNA-sprayed leaves was statistically significant (*P < 0.05; Student´s t test). (TIF) [file ppat.1005901.s003.tif]

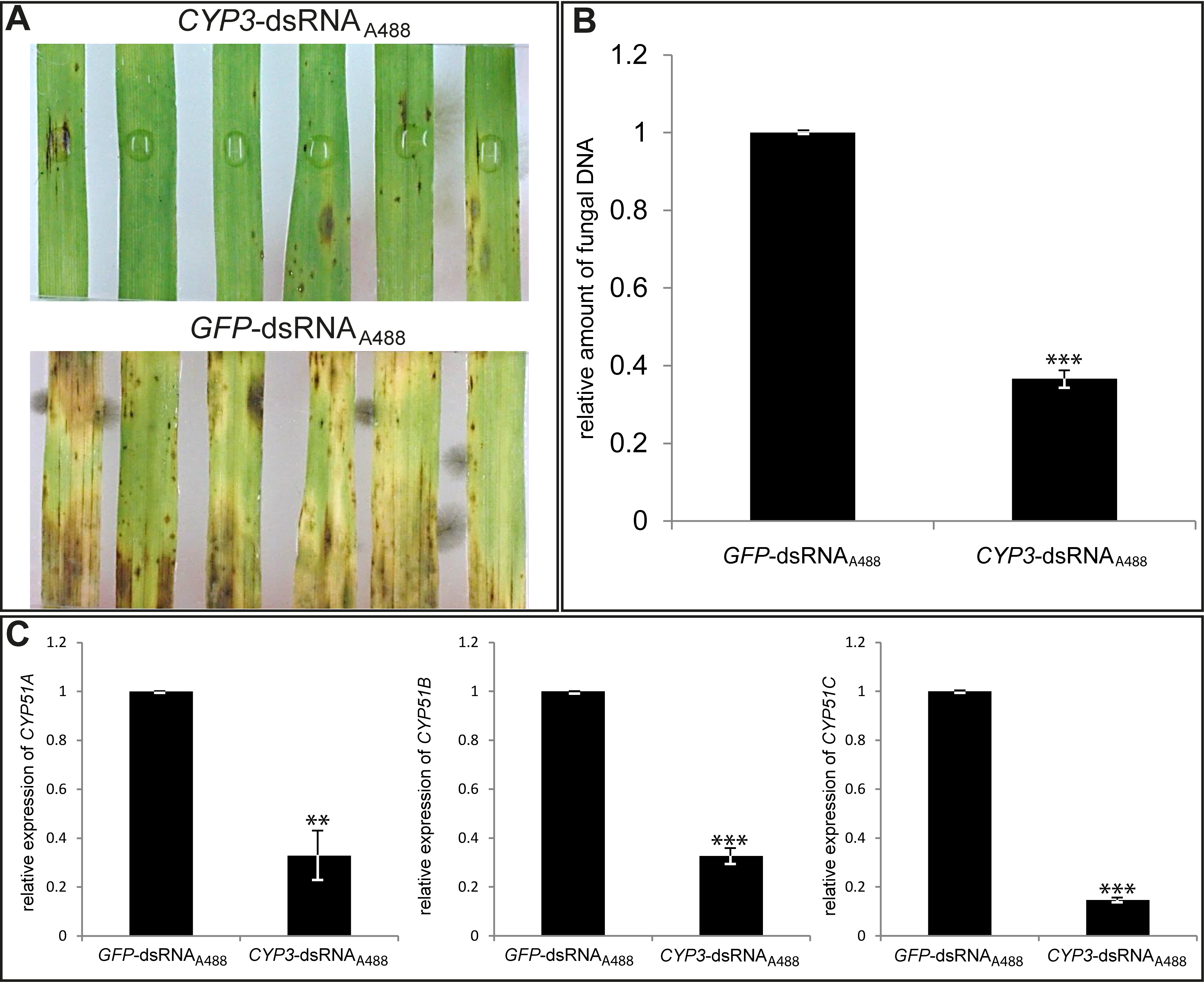

Supplement: S4 Fig — (A) F. graminearum infections are reduced in the distal tissue of leaves that received a 48 h pretreatment with CYP3-dsRNAA488 as compared to GFP-dsRNAA488 (control). (B) Quantification of fungal infection by qPCR. (C) Gene-specific quantification of CYP51 transcripts by qPCR. (TIF) [file ppat.1005901.s004.tif]

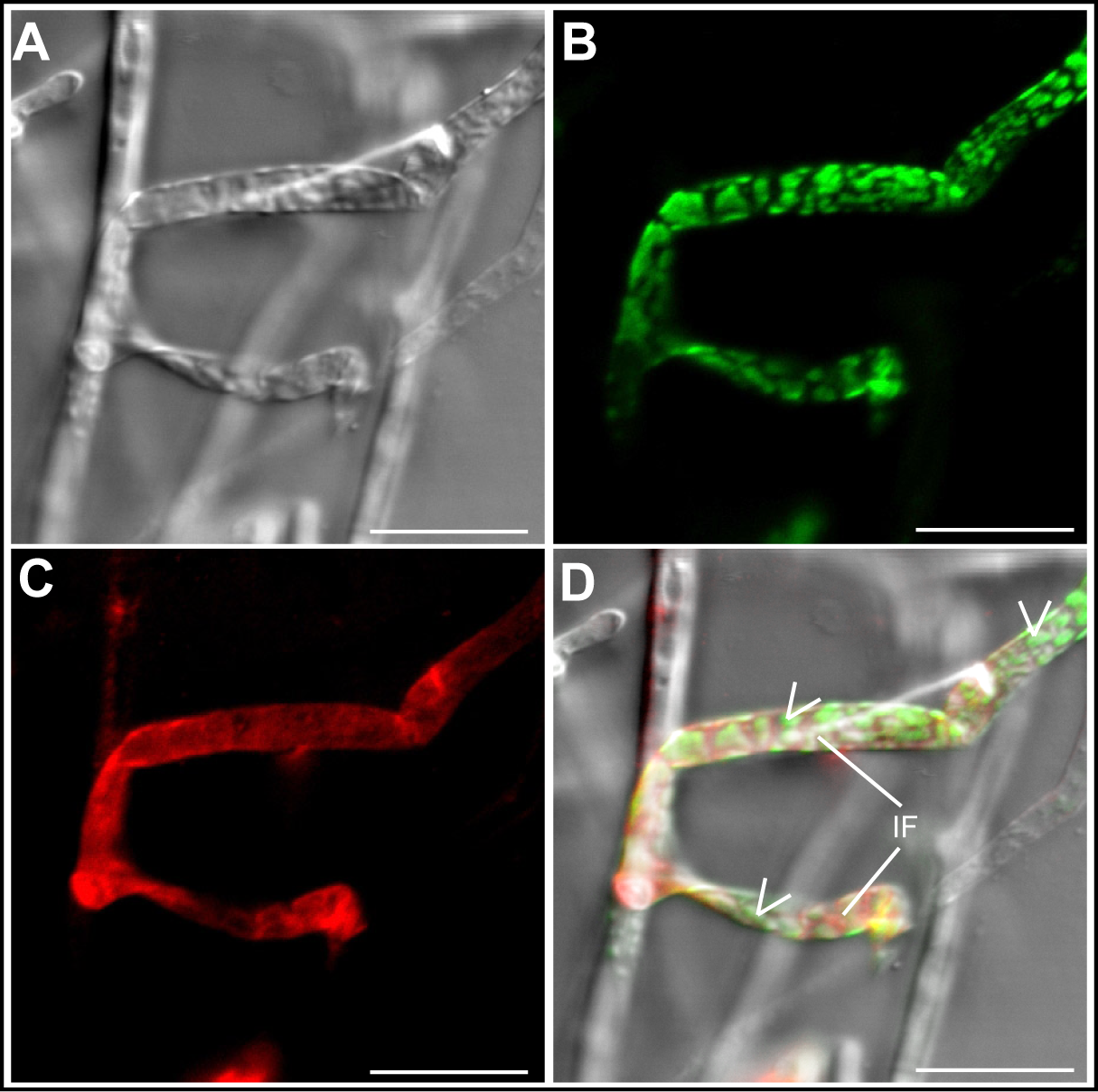

Supplement: S5 Fig — (A) Bright field microscopy of a fungal hyphae. (B) Hyphae strongly accumulated CYP3-dsRNAA488. (C) Hyphae stained with chitin-specific dye WGA-Alexa Fluor 594 (red). (D) Merge of B and C. RNA signals in germinated conidia are marked by arrow heads. Fungal infection hyphae (IF). Scale bars 10 μm. (TIF) [file ppat.1005901.s005.tif]

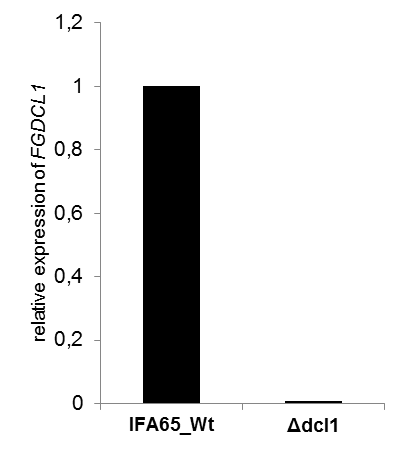

Supplement: S6 Fig — (TIF) [file ppat.1005901.s006.tif]

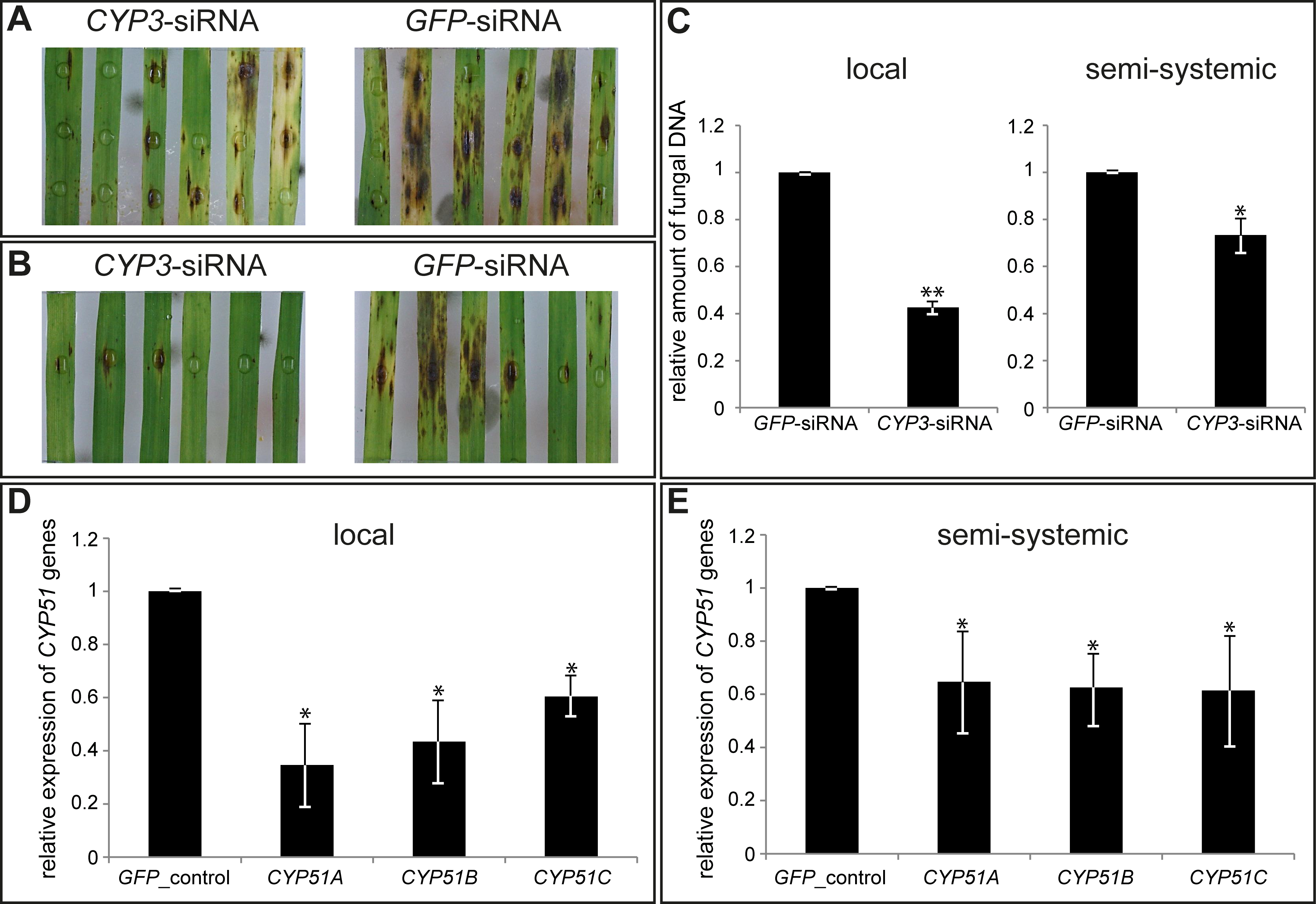

Supplement: S7 Fig — (A,B) Detached second leaves of three-week-old barley were sprayed with 20 ng μL-1 of CYP3-dsRNA-derived sRNA (CYP3-siRNA) or GFP-dsRNA-derived sRNA (GFP-siRNA, control) (see online methods), and 48 h later drop-inoculated with Fg-IFA65. Infection symptoms were evaluated at 6 dpi in the local (sprayed) (A) and distal, semi-systemic (non-sprayed) tissue (B). (C) qPCR quantification of fungal DNA in local and distal leaf tissues after spray-application of CYP3-siRNA or GFP-dsRNA at 6 dpi. (D,E) Gene-specific quantification of CYP51 transcripts by qPCR in local (D) and distal tissue (E) at 6 dpi. Bars represent mean values ± SDs of two independent experiments. (TIF) [file ppat.1005901.s007.tif]

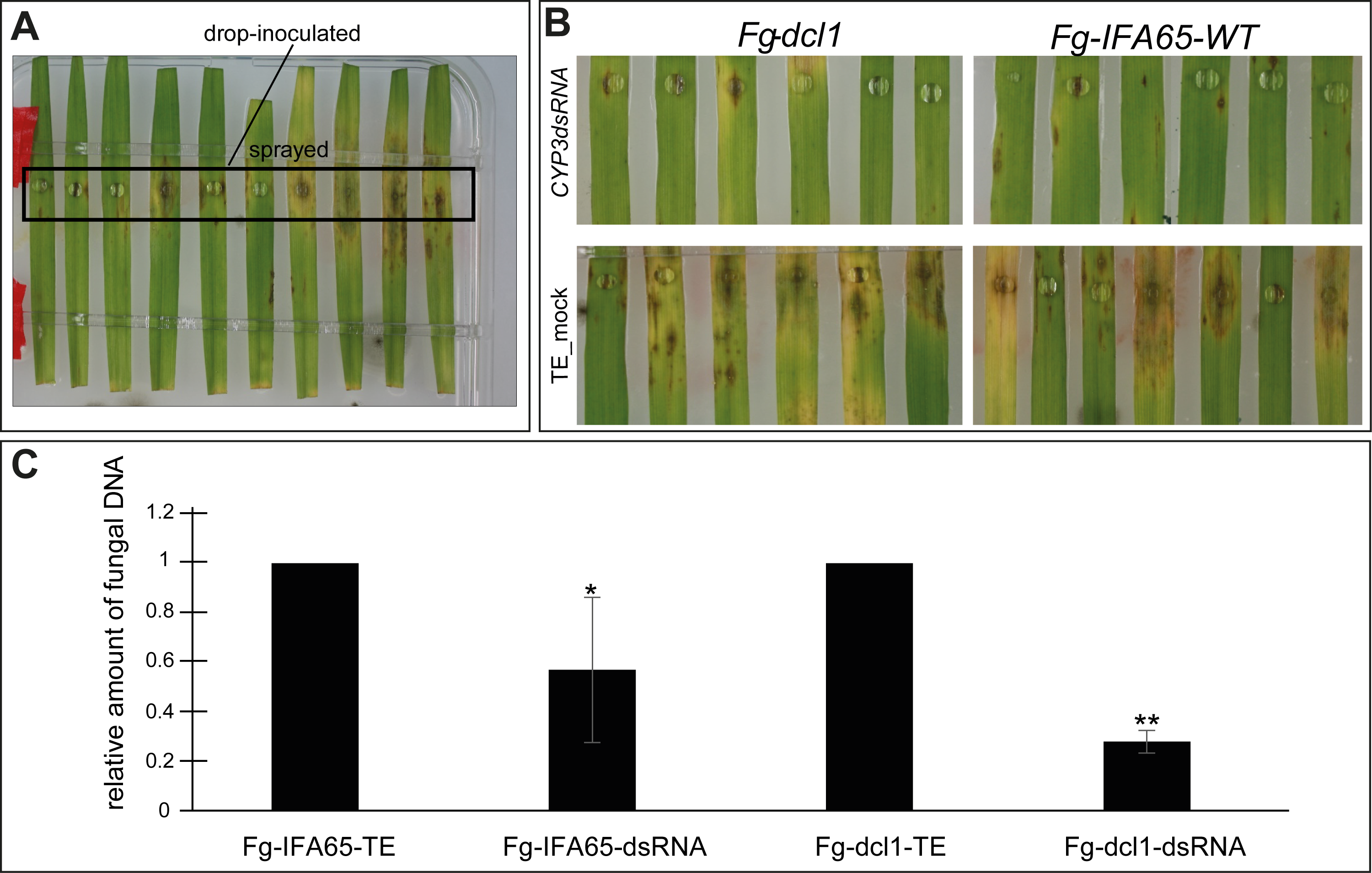

Supplement: S8 Fig — (A) Experimental design: Fusarium was drop-inoculated to the sprayed leaf area. (B) Both the fungal dicer-like-1 mutant Fg-IFA65Δdcl-1 and the wt strain (Fg-IFA65) were inhibited by CYP3-dsRNA as compared to TE treatment. Photographs were taken at 6 dpi. (C) Quantification of fungal DNA by qPCR analysis confirmed the macroscopic analysis. Bars represent mean values ±SDs of two independent sample collections. The reduction of fungal growth in samples treated with CYP3-dsRNA compared with mock-treated TE controls was statistically significant (*P < 0.05, **P < 0.01; Student´s t test). (TIF) [file ppat.1005901.s008.tif]
