# Supplementary material for: An RNAi-Based Control of Fusarium graminearum Infections Through Spraying of Long dsRNAs Involves a Plant Passage and Is Controlled by the Fungal Silencing Machinery
Source: PLoS Pathog. 2016 Oct 13;12(10):e1005901. doi: 10.1371/journal.ppat.1005901 (PMC5063301; doi:10.1371/journal.ppat.1005901)
Supplement: S1 Table — (DOCX) [file ppat.1005901.s009.docx]

**Supplementary Tab. 1** List of primers used in this study

| **Nr.** | **Primer name** | **Primer sequence** | **Application** |
| --- | --- | --- | --- |
| 1 | CYPBT7_F | TAATACGACTCACTATAGGGCAGCAAGTTTGACGAGTCCC | dsRNA synthesis |
|  | CYPCT7_R | TAATACGACTCACTATAGGGCATTGGAGCAGTCATAAACAACC | dsRNA synthesis |
| 2 | GFPT7_F | TAATACGACTCACTATAGGGGTGAGCAAGGGCGAG | dsRNA synthesis |
|  | GFPT7_R | TAATACGACTCACTATAGGGTTGTACAGCTCGTCCAT | dsRNA synthesis |
| 3 | CYP51A4_F | CCTTTGGTGCCGGTAGACAT | qRT-PCR |
|  | CYP51A4_R | CCCATCGAATAAACGCAGGC | qRT-PCR |
| 4 | QCYP51B_F | TCTACACCGTTCTCACTACTCC | qRT-PCR |
|  | QCYP51B_R | GCTTCTCTTGAAGTAATCGC | qRT-PCR |
| 5 | CYP51C2_F | CGAGTCCCTGGCACTGAATG | qRT-PCR |
|  | CYP51C2_R | GCTCATCACCCCAAAACCGT | qRT-PCR |
| 6 | ß-tubulin_F | ATCTCGAGCCCGGTACCATGG | qRT-PCR |
|  | ß-tubulin_R | CTCGGTGTAATGACCCTTGGCC | qRT-PCR |
| 7 | plantUbi_F | ACC CTC GCC GAC TAC AAC AT | qRT-PCR |
|  | plantUbi _R | CAG TAG TGG CGG TCG AAG TG | qRT-PCR |
| 8 | Fg-GFP_F | TTCACCTACGGCGTGCAGTGCTTCAGCC | qRT-PCR |
|  | Fg-GFP_R | CACCAGGGTGTCGCCCTCGAACTTCACC | qRT-PCR |
| 9 | PR1_F | GGACTACGACTACGGCTCCA | qRT-PCR |
|  | PR1_R | GGCTCGTAGTTGCAGGTGAT | qRT-PCR |
| 10 | JMT_F | TGACTTCCCCAAAATGAAGG | qRT-PCR |
|  | JMT_R | CTTCCGAGAAACAGCTGAGG | qRT-PCR |
| 11 | DCL_1_USS_KpnI _F | GGTACCAAAACCTGAGGTTACCATGC | USS/DSS cloning primers |
|  | DCL_1_USS_KpnI _R | GGTACCATTGAGGCTGTATTTGTTCT | USS/DSS cloning primers |
| 12 | DCL-1-DSS-HindIII_F | AAGCTTGTACTGCAGGGTATTTAGTGCACA | USS/DSS cloning primers |
|  | DCL-1-DSS-HindIII_R | AAGCTTGAATAAAAGACAACAACAGAAGGA | USS/DSS cloning primers |
| 13 | IFADicer_qpcr1_F | CCACGAGTTTAGGGCGATCA | qRT-PCR |
|  | IFADicer_qpcr1_R | TGCTGTTGGAAATGGGACTCA | qRT-PCR |
